# Adiponectin/resistin levels and insulin resistance in children: a four country comparison study

**DOI:** 10.1186/1687-9856-2015-2

**Published:** 2015-01-15

**Authors:** Koji Takemoto, Richard J Deckelbaum, Isao Saito, Supawadee Likitmaskul, Anita Morandi, Leonardo Pinelli, Eiichi Ishii, Kaichi Kida, Marwah Abdalla

**Affiliations:** Department of Pediatrics, Ehime University Graduate School of Medicine, Shitsukawa, Toon, Ehime 791-0295 Japan; Institute of Human Nutrition, Columbia University, New York, USA; Department of Pediatrics, Columbia University, New York, USA; Basic Nursing and Health Science, Ehime University Graduate School of Medicine, Toon, Japan; Department of Pediatrics, Faculty of Medicine, Siriraj Hospital, Mahidol University, Bangkok, Thailand; Centre for Pediatric Diabetes, Clinical Nutrition and Obesity, U.L.S.S. 20 and University of Verona, Verona, Italy; Division of Cardiology, Department of Medicine, Columbia University Medical Center, New York, USA

**Keywords:** Adiponectin, Resistin, Insulin resistance, Metabolic syndrome

## Abstract

**Background:**

There are few reports on the effects of ethnicity or gender in the association between adipocytokines and insulin resistance in children of different ages. This study assessed associations between serum concentrations of adiponectin/resistin and parameters of insulin resistance in children from 4 different countries.

**Methods:**

A total of 2,290 children were analyzed in this study; each was from one of 4 different countries (Japan, Thailand, Italy and USA), and grouped according to age (8–11 years old in Group 1 and 12–15 years old in Group 2).

**Results:**

Adioponectin was higher in female than in male children, and in Group 1 than in Group 2. Generally, adiponectin was lower in Asian as compared to Italian and American children. These tendencies remained even after adjustment for body mass index (BMI) or waist circumstance (WC). Among older children (Group 2), resistin was higher in female than in male children. Significant correlations by non-parametric univariate correlation coefficients and Spearman’s rank correlation coefficients were found between adiponectin and homeostasis model assessment of insulin resistance (HOMA-IR), and fasting serum insulin levels in young Japanese, Italian, and American female children(p < 0.01, p < 0.05, p < 0.05, respectively). Correlations between serum adiponectin and HOMA-IR were also found among older male Italian, American, and Thai children (p < 0.05, p < 0.001, p < 0.001, respectively). In multiple regression analysis by forced entry method, adiponectin correlated with HOMA-IR in Italian and American male children, and in all older female children regardless of country of origin. There was no correlation between resistin and markers of insulin resistance in children from any of the countries.

**Conclusions:**

We conclude that serum adiponectin concentrations are lower in Asian as compared to Italian and American children, and that adiponectin but not resistin contributes to differences in markers for insulin resistance in children from different populations.

## Background

Worldwide, there is an increase in the prevalence of obesity, insulin resistance, and metabolic syndrome among children, particularly within Asian countries [1]. As these children mature into adults, this raises concerns regarding their future risk of insulin resistance, type 2 diabetes, and cardiovascular diseases (CVD). Although obesity is a risk factor for insulin resistance and type 2 diabetes, not all obese people are insulin resistant and individuals have varying levels of insulin resistance for the same level of obesity [2]. For example, in certain ethnic groups in Singapore, adults of Asian origin are at an increased risk of insulin resistance and type 2 diabetes, despite having lower levels of obesity compared to adults of European and/or American origin [2]. One mechanism that has been postulated that contributes to ethnic variance in insulin resistance and development of type 2 diabetes are differences in adipocytokines.

It has been shown that the adipocytokines, adiponectin and resistin, can be predictors of all cause mortality in diabetics especially after myocardial infarction [3, 4]. Adiponectin has an important role in the development of metabolic syndrome [5, 6]. Nishimura et al. [5] showed that adiponectin concentrations were lower in obese children and adolescents and significantly associated with higher body mass index (BMI) in children. Winer et al. [6] reported that adiponectin in obese children was strongly associated with markers of insulin resistance and of inflammation such as C-reactive protein, but this latter association was independent of insulin resistance.

Previous studies with children and adolescents have reported strong correlations between adiponectin and markers of insulin resistance, such as fasting serum insulin or homeostasis model assessment of insulin resistance (HOMA-IR) [7, 8]. Kowalska et al. [7] reported that decreases in adiponectin concentrations and increases in fasting insulin were greater in individuals who met higher numbers of criteria for the metabolic syndrome according to National Cholesterol Education Program criteria [9]. Punthakee et al. [8] reported that adiponectin interacted with BMI Z-scores and that adiponectin correlated with insulin resistance in multiple regression analyses.

In adults, adiponectin concentrations vary by ethnicity. Whether effects of ethnicity on adiponectin are independent of obesity and insulin resistance remains unclear in pediatric populations [2]. Understanding the relationship between adiponectin, insulin resistance, and ethnicity may provide an insight into why certain ethnic groups may be at higher risk of insulin resistance and type 2 diabetes.

The relationship between resistin and insulin resistance is controversial. Osawa et al. [10] reported that single nucleotide polymorphisms (SNPs) in the resistin gene were strongly associated with insulin resistance in adults with type 2 diabetes. Yagmur et al. [11] showed that elevated resistin contributes to insulin resistance, particularly in patients with liver cirrhosis, but Li et al. [12] and de Luis et al. [13] found no correlation or only a weak correlation between resistin concentration and insulin resistance in children and adults.

To date, there are only a few reports on the effects of ethnicity or gender on the association between adipocytokines and insulin resistance in children and a lack of large population-based comparative studies among children of different ages [14, 15]. The largest and most recent study is the Child Heart and Health Study (CHASE) which included a multiethnic cohort of 4, 633 9–10 year old British children from South Asian, Afro-Caribbean, and European origins [16]. Among this cohort, British children of South Asian descent had the strongest association between adioposity and HOMA-IR. However, adiponectin and resistin were not assessed in this population.

The aim of our population-based comparison study was to investigate the potential association between insulin resistance and adiponectin and resistin, in young and older children from 4 countries, Japan, Thailand, Italy and the USA. Our hypotheses were that adiponectin and resistin concentrations would vary by age, BMI, gender, and country of origin. Specifically, because of higher predicted prevalence of overweight and obesity and higher insulin resistance, we hypothesized that older US and Italian children would have higher BMIs, lower adiponectin, and higher resistin compared to Japanese and Thai children.

## Methods

### Participants

A total of 2,290 healthy children from Japan, Thailand, Italy and USA were enrolled in this study. Children were assigned to 1 of 2 groups according to their age (Group 1 children were aged 8–11 years old and Group 2 children were aged 12–15 years old).

In Matsuyama, Japan, all school children aged 9–10 and 12–13 years (approximately 10,000 children) have undergone annual screening for serum cholesterol, blood pressure, and obesity in a special school program beginning in 1989. For the current study, we randomly enrolled 934 children (752 in Group 1 and 182 in Group 2) from this program in 2002. In Bangkok, Thailand, 472 students (247 boys from an all-boys school and 225 girls from an all-girls school) aged 12–13 years with Thai or Thai-Chinese ethnicity were enrolled in Group 2 in 2002; there were no children enrolled in Group 1 from Thailand. Because of the initial design of the school based program in Thailand and Japan, participants did not undergo pubertal screening at the time of enrollment and thus information regarding Tanner sexual maturity stage was not obtained. In Verona, Italy, 4,000 children aged 9–13 years were randomly selected in 2003 from the database of the city’s registry office, and their families were invited to join the study by mail. Children from the first 700 families, who called the study’s free-dial telephone number, were enrolled. The study design did not include information on Tanner sexual maturity stage. In the USA, we enrolled children who participated in the Columbia University BioMarkers Study [17], a cross-sectional study of American children and their parents conducted between 1994 and 1997. The BioMarkers Study recruited 1,054 children with a mean age of 9.9 years. Data for these analyses includes a sub-group of participants who originally enrolled in the study in 1994–1997 of which 188 were included in this study (98 in Group 1, 90 in Group 2). Initial biomarker data was collected and stored at −70 C for future secondary analyses. At the time of initial study design, data on Tanner sexual maturity stage was not obtained. The ethnicity/race of children were recorded according to the mother’s self-report following definitions used in the 1990 US census which classified individuals as “Hispanic”, “White but not of Hispanic origin”, “Black but not of Hispanic origin”, and “Asian or Pacific Islander”. Most Biomarker Study children were Caucasian or Hispanic. Although children and adults aged 4–25 years were eligible to participate in the BioMarkers Study; only children aged 8–11, and 12–15 years were included in this subsequent comparative study. To be eligible for study enrollment, children had to be healthy, and those with renal, cardiovascular, or genetic syndromes/diseases, as well as those who were unwilling (themselves or their parents) to participate in the study, were excluded. Those with missing baseline, anthropometric, or lab values were also excluded. The number of male and female children included in the different groups were as follows: from Japan, 410 male and 342 female children were included in Group 1, and 97 male and 85 female children in Group 2; from Thailand, 247 male and 225 female children were included in Group 2; no children were enrolled in Group 1; from Italy, 220 male and 164 female children were included in Group 1, and 168 male and 144 female children in Group 2; from the USA, 55 male and 43 female children were included in Group 1, and 45 male and 45 female children in Group 2. All participants were from self-identified middle income socioeconomic families as defined by the median family income in each respective country.

The Institutional Review Boards of each of the participating institutions in each of the 4 countries approved the protocols. Before the study commenced, written informed consent and assent to participate were obtained from the parents and children, respectively.

### Methods

We used waist circumstances (WC), systolic and diastolic blood pressure (BP), triglyceride (TG) and high-density lipoprotein-cholesterol (HDL-C) and fasting plasma glucose as the parameters of metabolic syndrome in children [18] and fasting serum insulin levels and HOMA-IR as the parameters of insulin resistance [19]. Risk factors associated with the metabolic syndrome followed criteria of the International Diabetes Federation (IDF) [18]. In Italy, BMI was used rather than the data of WC because of lack of data on WC.

Anthropometric parameters included height, body weight, WC, BMI and BMI Z-scores were calculated in all countries. Height was measured using a portable standiometer (Leicester Height Measure; Invicta Plastics Ltd., Oadby, England) in Japan and Thailand. In Italy, height was measured to the nearest 0.5 cm on a standardized height board. In the US, height was measured using a rigid standiometer to the nearest centimeter.

Weight and percent body fat was measured by the impedance method (TBF-300A, Tanita Co., Tokyo, Japan in Japan, Thailand and Italy. In USA, body weight was measured to the nearest 0.1 kg using a calibrated triple beam-balance scale. Percent body fat data was not measured in the USA. In all countries BMI was calculated as the weight in kilograms divided by the height in meters squared.

In Japan, WC was measured in the standing position at the level of the umbilicus at end-expiration. In Thailand, WC was measured by tape midway between the bottom of the lower rib and the iliac crest while the subject was standing. In the USA, WC (defined as the narrowest part of the torso) was determined by tape measure. WC data was not collected in Italy.

BP measurement assays varied. In Japan, BP was measured while seated. If the systolic or diastolic BP was at a hypertensive level (systolic BP ≥ 135 mmHg, diastolic BP ≥80 mmHg in elementary and junior high school females, and systolic BP >140 mmHg, diastolic BP ≥85 mmHg in junior high school males, BP was measured twice more and mean levels were calculated. In Thailand, BP was measured twice by an automatic blood pressure monitor model T4 (Omron Corporation; Tokyo, Japan) while seated. In Italy, BP was measured using an automated oscillometric device (Digital Blood Pressure Monitor HEM-907, OMRON, Kyoto, Japan) by trained medical staff using standard protocols [20]. Following an initial 10-minute period of rest, three seated readings were obtained at 1-minute intervals. The mean BP was determined as the mean of the three readings. In the USA, BP was measured using an automated oscillometric device (Dinemap 8100 vital signs monitor; Critikon, Inc., Tampa, FL, USA) by trained medical staff using standard protocols [20]. Following an initial 5-minute period of rest, five seated readings were obtained at 1-minute intervals. The mean BP was determined as the mean of readings two through five.

We collected fasting blood samples after an overnight fast of more than 10 hours. Samples were separated into serum and/or plasma and were frozen. Samples from all countries were sent on dry ice and analyzed by the same methodologies in one laboratory at SRL Laboratory (Tokyo, Japan). Triglyceride (TG, L-Type Triglyceride H, Wako) levels were analyzed using enzymatic methods. High-density lipoprotein-cholesterol (HDL-C) (Cholestest N HDL, Daiichi) was analyzed using a synthetic polymer method. Serum adiponectin, and resistin levels were measured using an enzyme-linked immunosorbant assay (Human Adiponectin ELISA Kit, Otsuka Pharmaceutical Co., Tokyo, Japan), and an immunoassay (Human Resistin Immunoassay, R&D Systems Inc., Minneapolis, MN, USA), respectively. Serum total adiponectin levels include both high and low molecular weight adiponectin, but these were not separately analyzed. HOMA-IR was determined from fasting plasma glucose levels, which were measured enzymatically (Sik Liquid GLU, Kanto Chemical Co., Inc., Tokyo, Japan) and fasting serum insulin levels, which were measured using a chemiluminescent enzyme immunoassay (LUMIPULSE Presto Insulin, Fujirebio Inc., Tokyo, Japan). HOMA-IR was defined as multiplying fasting plasma glucose levels (mg/dL) by fasting serum insulin levels (μU/mL) times 1/405. Analyses were performed by SRL, Inc. between 2003 and 2006.

### Statistical analysis

Data are presented as means ± standard error (SE). Analysis of variance (ANOVA) was used to compare concentrations of adipocytokine among children from different countries. Student’s *t*-tests were used to compare adipocytokines between male and female children and between Group 1 and Group 2. Non-parametric univariate correlation coefficients and Spearman’s rank correlation coefficients were used to ascertain relationships between adiponectin, resistin, and other parameters. Multiple regression analyses were performed with fasting serum insulin levels or HOMA-IR as the dependent and serum adiponectin, resistin, and other factors of metabolic syndrome as independent variables with forced entry approaches. Differences were considered statistically significant at p < 0.05.

## Results

The baseline clinical characteristics of the children from all 4 countries, including anthropometric parameters, BP, insulin resistance, and lipid and adipocytokine levels for Group 1 and Group 2 are shown in Tables 1 and 2, respectively. As shown in the table differences in anthropometrics, BP, and lipid levels were found among the groups in the different countries. Across all age groups and genders, American children had higher weights, systolic/diastolic BPs, higher triglycerides, and lower HDL levels when compared to children from the other countries (p < 0.05).

**Table 1:** **Baseline clinical characteristics of group 1 children from Japan, Italy, and the USA**

	Males			Females		
	Japan	Italy	USA	Japan	Italy	USA
n	411	220	55	341	164	43
Age	9.5 ± 0.0	9.4 ± 0.0	9.2 ± 0.1	9.6 ± 0.0	9.6 ± 0.0	9.6 ± 0.2
Height (cm)	135.4 ± 0.3^a,b^	138.3 ± 0.5	138.0 ± 1.3	135.9 ± 0.4^a,b^	138.8 ± 0.6^c^	143.2 ± 1.4
Weight (kg)	32.8 ± 0.4^a,b^	34.8 ± 0.5^c^	41.0 ± 1.9	32.1 ± 0.4^a,b^	34.5 ± 0.5^c^	43.2 ± 1.9
BMI	17.7 ± 0.2^b^	18.0 ± 0.2^c^	21.1 ± 0.7	17.2 ± 0.1^a,b^	17.9 ± 0.2^c^	20.8 ± 0.7
Waist (cm)	60.6 ± 0.9	NT	68.6 ± 12.0	58.2 ± 0.4	NT	67.1 ± 12.1
Systolic B.P. (mmHg)	108 ± 0.7^a,b^	99.0 ± 0.9^c^	113 ± 1.5	109 ± 0.7^a,b^	98 ± 0.8^c^	115 ± 1.4
Diastolic B.P. (mmHg)	58 ± 0.4	58 ± 0.8	60 ± 1.2	58 ± 0.4^b^	59 ± 0.8^c^	64 ± 1.2
HDL-C (mg/dl)	66.5 ± 0.6^a,b^	61.5 ± 0.8^c^	45.1 ± 1.5	65.0 ± 0.6^a,b^	60.2 ± 0.8^c^	48.3 ± 3.5
TG (mg/dl)	67.1 ± 2.2^a,b^	52.8 ± 2.1^c^	105.0 ± 6.5	75.8 ± 2.3^a,b^	61.7 ± 2.2^c^	116.9 ± 7.6
Insulin (μU/ml)	6.1 ± 0.6^a,b^	9.6 ± 0.4	11.2 ± 2.0	7.9 ± 0.5^a,b^	12.0 ± 0.5^c^	18.0 ± 2.5
Glucose (mg/dl)	91.1 ± 0.5^a^	87.9 ± 0.6	90.1 ± 0.9	90.3 ± 0.5^b^	86.7 ± 0.5	89.1 ± 1.5
HOMA-IR	1.41 ± 0.15^a,b^	2.11 ± 0.10	2.51 ± 0.45	1.79 ± 0.11^a,b^	2.59 ± 0.12^c^	4.16 ± 0.6
Adiponectin (μg/ml)	12.0 ± 0.4^a,b^	14.0 ± 0.4	14.0 ± 0.7	13.2 ± 0.4^a^	14.9 ± 0.4	14.6 ± 1.0
Resistin (μg/ml)	9.1 ± 0.5	10.1 ± 0.3^c^	8.4 ± 0.3	10.6 ± 0.5	10.8 ± 0.3	9.1 ± 0.6

Each value indicates mean ± S.E. (standard error).

Analysis of variance (ANOVA) was used to compare all parameters among children from different countries.

The a, b, c mean p < 0.05 between Japan and Italy (a), Japan and USA (b), and Italy and USA (c).

BMI, body mass index; B.P., blood pressure; HDL-C, high-density lipoprotein-cholesterol; TG, triglycerides; HOMA-IR, homeostasis model assessment of insulin resistance; NT, not tested.

**Table 2:** **Baseline clinical characteristics of group 2 children from Japan, Thailand, Italy, and the USA**

	Males				Females			
	Japan	Thailand	Italy	USA	Japan	Thailand	Italy	USA
n	97	247	168	45	85	223	144	45
Age	12.5 ± 0.1	13.1 ± 0.0	12.3 ± 0.0	13.1 ± 0.2	12.6 ± 0.1	13.0 ± 0.0	12.3 ± 0.0	13.3 ± 0.2
Height (cm)	155.6 ± 0.8^b,c^	159.2 ± 0.5	154.9 ± 0.7^d^	159.6 ± 2.0	153.2 ± 0.6^c^	154.0 ± 0.4	154.9 ± 0.6	156.5 ± 1.0
Weight (kg)	45.6 ± 1.0^b,c^	55.3 ± 0.9	48.9 ± 0.9^d,e^	58.1 ± 2.6	44.4 ± 1.0^b,c^	49.9 ± 0.9^f^	46.7 ± 0.8^e^	58.4 ± 2.5
BMI	18.7 ± 0.3^a,b,c^	21.8 ± 0.3	20.2 ± 0.3^d,e^	22.4 ± 0.7	18.8 ± 0.3^b,c^	21.0 ± 0.3^f^	19.3 ± 0.2^e^	23.6 ± 0.9
Waist (cm)	66.4 ± 0.8	76.8 ± 13.0	NT	68.6 ± 12.0	61.8 ± 1.0	68.4 ± 10.0	NT	67.1 ± 12.1
Systolic B.P. (mmHg)	112 ± 1.5^b,c^	116 ± 0.8^f^	110. ± 1.0^d,e^	122 ± 1.4	109 ± 1.5^c^	111 ± 0.7	108 ± 1.2^e^	115 ± 1.5
Diastolic B.P. (mmHg)	60 ± 0.8^b,c^	68 ± 0.6^f^	60 ± 0.7^d,e^	64 ± 1.1	59 ± 0.9^a,b^	69 ± 0.5^f^	62 ± 0.9^d^	62 ± 1.0
HDL-C (mg/dl)	64.6 ± 1.4^b,c^	60.6 ± 0.8^f^	60.9 ± 0.9^e^	41.8 ± 1.2	64.0 ± 1.3^a,b,c^	60.4 ± 0.8^f^	59.5 ± 0.8^e^	42.2 ± 1.5
TG (mg/dl)	57.4 ± 3.4^a,b,c^	73.5 ± 2.8^f^	57.3 ± 2.6^d,e^	105.0 ± 6.5	69.4 ± 3.3^c^	70.8 ± 1.8^f^	66.1 ± 2.3^e^	111.9 ± 7.7
Insulin (μU/ml)	8.3 ± 0.6^a,c^	10.1 ± 0.7^f^	12.8 ± 0.8^d^	14.3 ± 1.4	9.0 ± 0.6^a,c^	9.0 ± 0.5^f^	15.5 ± 0.6^d,e^	23.1 ± 2.8
Glucose (mg/dl)	92.8 ± 0.6	92.6 ± 0.4	90.5 ± 6.0^d^	91.7 ± 1.2	92.9 ± 0.7^a,b,c^	89.5 ± 0.5	89.5 ± 0.5	89.2 ± 1.0
HOMA-IR	1.95 ± 0.15^a,c^	2.35 ± 0.18^f^	2.91 ± 0.19	3.28 ± 0.33	2.10 ± 0.15^a,c^	2.03 ± 0.13^f^	3.45 ± 0.14^d,e^	5.26 ± 0.7
Adiponectin (μg/ml)	10.7 ± 0.5^a^	10.2 ± 0.4^f^	12.8 ± 0.4^d^	13.0 ± 1.0	11.0 ± 0.4^a,c^	12.0 ± 0.4	13.6 ± 0.4^d^	13.2 ± 1.0
Resistin (μg/ml)	14.8 ± 0.8^a,b,c^	19.4 ± 1.3^f^	9.9 ± 0.3^d^	9.1 ± 0.6	16.4 ± 1.2^a,c^	20.4 ± 1.6^f^	11.3 ± 0.4^d^	10.3 ± 0.6

Each value indicates mean ± S.E. (standard error).

Analysis of variance (ANOVA) was used to compare all parameter levels among children from different countries.

The a, b, c, d, e, f mean p < 0.05 between Japan and Italy (a), Japan and Thailand (b), Japan and USA (c), Italy and Thailand (d), Italy and USA (e), and Thailand and USA (f).

BMI, body mass index; B.P., blood pressure; HDL-C, high-density lipoprotein-cholesterol; TG, triglycerides; HOMA-IR, homeostasis model assessment of insulin resistance; NT, not tested.

*Parameters of Insulin Resistance:*

- Age Differences: In children from Japan, Italy, and the USA, fasting glucose levels, insulin, and HOMA-IR values were higher in older Group 2 than in Group 1 children (p < 0.05).
- Gender Differences: When compared to males within Japan, Italy, and the USA and across age groups, females had higher levels of insulin and HOMA-IR (p < 0.05). Interestingly, Thai females had lower insulin and HOMA-IR values when compared to Thai male children (p < 0.05).
- Ethnic Differences: Among both genders, serum insulin levels and HOMA-IR were significantly higher in children from Italy and the USA compared to children from Japan in Group 1. In Group 2, Italian and American children also had higher insulin and HOMA-IR levels compared with Japanese and Thai children. Japanese children had the highest fasting glucose levels (p < 0.05).

*Adiponectin:*

**Figure 1:**
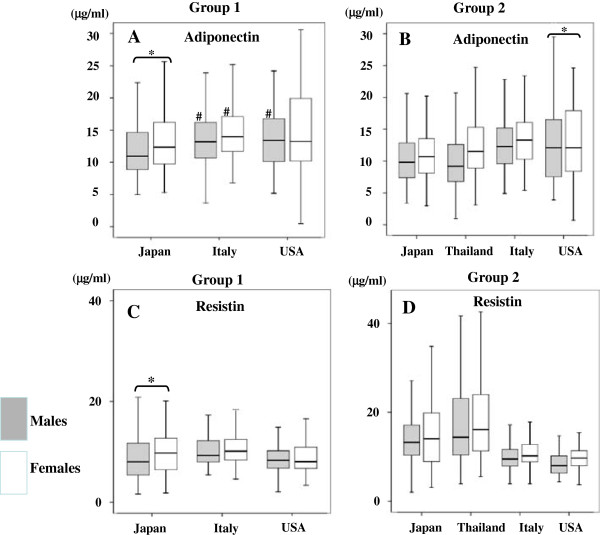
**Serum adiponectin (A and B) and resistin (C and D) concentration in children divided into Groups 1 and 2 according to sex and country of origin. A**: Serum adiponectin concentration in Group 1, **B**: Serum adiponectin concentration in Group 2, **C**: Serum resistin concentration in Group 1, **D**: Serum resistin concentration in Group 2. White squares indicate female children, and gray squares indicate male children. #p < 0.05 indicate significant levels in children from Japan compared to those from other countries. *  *p < 0.05 indicate significant differences in male children compared to female children. Analysis of variance (ANOVA) was used to compare adipocytokine levels among children from different countries. Student’s *t*-tests were used to compare adipocytokine levels between male and female children and between Group 1 and Group 2.

- Age Differences: Across all countries, serum adiponectin concentration was higher in younger Group 1 than in older Group 2 children (Tables 1 and 2).b) Gender Differences: Figure 1 shows sex-based differences in serum adiponectin concentration stratified by age and ethnicity. Overall, serum adiponectin levels were significantly higher or tended to be higher in female children than in male children. In Group 1 children, Japanese male children consistently had the lowest serum adiponectin concentration compared with male children from Italy and the USA. Japanese males also had significantly lower adiponectin compared with Japanese females. However, in the other countries, there were no significant sex-based differences in adiponectin levels among Group 1 children (Figure 1A). In Group 2 children, adiponectin was significantly lower in both male and female Japanese children compared with all other countries. American females had higher adiponectin compared with American males (Figure 1B). These tendencies remained seen after the adjustment for BMI or WC (data not shown).

c) Ethnic Differences: Italian females in both age groups had the highest adiponectin whereas Thai male children in Group 2 had the lowest adiponectin. Of note, Asian children had significantly lower or a trend for lower adiponectin than children from Italy or the USA (p < 0.05).

*Resistin:*

- Age Differences: In contrast to adiponectin, resistin was higher in older Group 2 children across all countries.b) Gender Differences: Figure 1C and D show serum resistin concentrations according to age, gender, and ethnicity. Females in both age groups had either significantly higher resistin than males, or showed a trend in the same direction. Serum resistin concentrations of younger Group 1 children from Japan, the USA, and Italy were overall similar (Figure 1C). However, there were sex-based differences among Japanese children in Group 1. Japanese male children had lower resistin compared with Japanese female children in this age group. Sex-based differences also existed among Group 2 Italian children. Italian females had higher resistin compared with Italian males.c) Ethnic Differences: Group 2 Japanese children had significantly higher serum resistin concentrations compared with Italian and American children (p < 0.05). American children had the lowest levels of resistin across all four countries while Thai Group 2 children had the highest resistin among all 4 countries (Figure 1D). These trends were seen independently of adjustments for BMI or WC (data not shown).

Table 3 shows the non-parametric univariate correlation coefficients and Spearman’s rank correlation coefficients between serum adiponectin and resistin levels and parameters that are associated with metabolic syndrome and insulin resistance. As expected, there were negative correlations between BMI and adiponectin. Serum adiponectin concentrations and HOMA-IR as well as fasting serum insulin were negatively correlated in Group 1 females from all countries and in Group 2 females from Japan, Thailand, and Italy. Among males, there were differences in correlation among the different age groups. Adiponectin and HOMA-IR were negatively correlated only among Thai and USA male children.

**Table 3:** **Correlation coefficients between adiponectin, resistin and selected parameters related to metabolic syndrome and insulin resistance**

Males	Group 1						Group 2							
	Adiponectin			Resistin			Adiponectin				Resistin			
	Japan	Italy	USA	Japan	Italy	USA	Japan	Thailand	Italy	USA	Japan	Thailand	Italy	USA
**BMI Z-scores**	−0.117	−0.143	−0.236	0.072	0.154	0.139	−0.339*	−0.455*	−0.243^*^	−0.487*	0.032	0.054	0.216^*^	−0.066
**Waist**	−0.121	-	−0.135	0.092	-	0.226	−0.3^*^	−0.454*	-	−0451^*^	0.035	0.081	-	−0.176
**Systolic B.P.**	−0.064	0.029	0.003	0.049	0.206^*^	0.289^*^	−0.213^*^	−0.206^*^	0.071	−0.303^*^	−0.231^*^	0.008	0.098	−0.156
**Diastolic B.P.**	−0.053	0.132	0.114	0.153	0.182*	0.126	−0.043	0.184*	0.005	0.229	−0.024	−0.006	−0.051	0.026
**HDL-C**	0.25^*^	0.417*	0.284^*^	−0.05	−0.157	−0.164	0.346*	0.389*	0.260^*^	0.282	0.013	−0.032	−0.083	−0.210
**TG**	−0.166	−0.215^*^	−0.347^*^	0.085	0.182^*^	0.253	−0.24^*^	−0.27^*^	−0.087	0.013	−0.082	−0.003	0.098	0.079
**Insulin**	−0.143	−0.088	−0.188	−0.001	0.122	0.147	−0.179	−0.394*	−0.196^*^	−0.475*	−0.094	0.049	0.088	−0.228
**HOMA-IR**	−0.157	−0.078	−0.207	−0.003	0.126	0.131	−0.189	−0.389*	−0.176	−0.471*	−0.116	0.028	0.110	−0.263
**Females**	**Group 1**						**Group 2**							
	**Adiponectin**			**Resistin**			**Adiponectin**				**Resistin**			
	**Japan**	**Italy**	**USA**	**Japan**	**Italy**	**USA**	**Japan**	**Thailand**	**Italy**	**USA**	**Japan**	**Thailand**	**Italy**	**USA**
**BMI Z-scores**	−0.196^*^	−0.196^*^	−0.498*	0.095	0.228^*^	0.224	−0.486*	−0.478*	−0.047	−0.303^*^	−0.018	0.289*	0.143	0.115
**Waist**	−0.263^+^	-	−0.483*	−0.003	-	0.174	−0.45*	−0.501*	-	−0.163	0.002	0.14	-	0.043
**Systolic B.P.**	−0.017	−0.023	−0.361^*^	0.209^*^	0.241^*^	−0.023	−0.232^*^	−0.384*	0.018	−0.011	−0.044	0.206^*^	0.165	0.031
**Diastolic B.P.**	0.224*	−0.031	−0.051	0.126	0.177	−0.165	−0.161	−0.136	−0146	−0.125	−0.027	0.106	0.123	0.047
**HDL-C**	0.234^*^	0.352*	0.213	0.106	−0.102	−0.049	0.293^*^	0.274^*^	0.113	0.414^*^	−0.069	−0.075	−0.166	0.100
**TG**	−0.207^*^	−0.105	0.014	−0.100	0.16	−0.162	−0.265^*^	−0.136	−0.2^*^	−0.02	0.09	0.007	0.073	0.025
**Insulin**	−0.239^*^	−0.212^*^	−0.33^*^	0.001	0.268^*^	−0.044	−0.386*	−0.478*	−0.217^*^	−0.076	0.133	0.112	0.106	−0.079
**HOMA-IR**	−0.247^*^	−0.199^*^	−0.346^*^	0.015	0.256^*^	0.001	−0.397*	−0.475*	−0.227^*^	−0.067	0.155	0.125	0.103	−0.117

Each value represents the correlation coefficient between two parameters. p-values are as follows; *p < 0.05.

BMI, body mass index; B.P., blood pressure; HDL-C, high-density lipoprotein-cholesterol; TG, triglycerides; HOMA-IR, homeostasis model assessment of insulin resistance.

In comparison, correlations between serum resistin and measured parameters were inconsistent. Serum resistin concentration did not correlate with HDL-C levels in any group or ethnicity. Serum resistin and systolic BP consistently correlated in Italian Group 1 and Group 2 male children. Serum resistin and BMI positively correlated only in certain groups such as Group 1 Italian females, in Group 2 Thailand females, and Group 2 Italian males. Likewise, serum resistin and HOMA-IR were positively correlated only among Group 1 Italian females.

In multiple regression analyses using forced entry methods, serum adiponectin levels were or tended to be associated with HOMA-IR in Group 2 male children from Italy and the USA and in Group 2 female children from all countries (Table 4). Resistin was associated with HOMA-IR in children from any of the countries (Table 4).

**Table 4:** **Regression coefficients between HOMA-IR and anthropometric parameters, serum lipids and adipocytokines in multiple regression analyses**

Males	Group 1						Group 2							
	Japan		Italy		USA		Japan		Thailand		Italy		USA	
	β	p	β	p	β	p	β	p	β	p	β	p	β	p
Waist **	0.464	<0.001*	0.293	0.01*	0.381	0.004	0.322	0.003^*^	0.365	<0.001*	0.578	0.006^*^	0.245	0.176
**Systolic B.P.**	0.162	0.041^*^	0.015	0.079	0.116	0.334	0.036	0.706	0.244	0.002^*^	0.046	0.002^*^	0.139	0.412
**TG**	0.140	0.086	0.003	0.294	0.394	0.007	0.443	<0.001*	0.234	0.005^*^	0.014	0.023^*^	−0.020	0.899
**HDL-C**	−0.128	0.128	−0.061	0.882	0.265	0.054	0.064	0.533	−0.007	0.928	0.003	0.844	−0.044	0.792
**adiponectin**	−0.029	0.708	−0.008	0.716	−0.082	0.504	0.019	0.836	−0.062	0.430	−0.083	0.04*	−0.291	0.078
**resistin**	−0.042	0.578	0.015	0.611	0.039	0.744	−0.112	0.197	−0.035	0.607	−0.064	0.312	−0.112	0.485
**Females**	**Group 1**						**Group 2**							
	**Japan**		**Italy**		**USA**		**Japan**		**Thailand**		**Italy**		**USA**	
	**β**	**p**	**β**	**p**	**β**	**p**	**β**	**p**	**β**	**p**	**β**	**p**	**β**	**p**
Waist **	0.318	<0.001*	0.337	0.009^*^	0.425	0.010^*^	0.094	0.367	0.343	0.001^*^	0.403	0.005^*^	0.347	0.028^*^
**Systolic B.P.**	0.153	0.072	0.007	0.544	0.151	0.321	0.278	0.012^*^	0.157	0.086	0.001	0.952	0.131	0.405
**TG**	0.264	0.002^*^	0.014	0.001*	0.206	0.207	0.092	0.396	0.126	0.102	0.013	0.012*	−0.180	0.249
**HDL-C**	−0.134	0.111	0.017	0.187	−0.074	0.645	−0.088	0.445	0.120	0.132	0.015	0.314	−0.455	0.006^*^
**adiponectin**	−0.082	0.321	−0.043	0.115	−0.187	0.202	−0.299	0.010^*^	−0.179	0.052	−0.071	0.025^*^	0.278	0.065
**resistin**	−0.034	0.680	0.025	0.424	−0.070	0.619	0.141	0.166	0.041	0.599	0.031	0.293	−0.103	0.461

Each value means the b- and p-value between HOMA-IR and each factor; *p < 0.05.

**In Italy, the data of BMI Z-scores was used rather than the data of waist.

BMI, body mass index; B.P., blood pressure; HDL-C, high-density lipoprotein-cholesterol; TG, triglycerides; HOMA-IR, homeostasis model assessment of insulin resistance.

## Discussion

Adiponectin is an adipocytokine that is closely associated with insulin resistance. Generally, insulin resistance is associated with lower serum adiponectin concentrations [21], and many studies have shown the strong inverse associations between adiponectin and insulin resistance or metabolic syndrome [22–24]. However, these findings stem from childhood obesity-related studies, and there are no or few comparable international studies that show relationships between adipocytokines and insulin resistance in children. The aim of our population-based comparison study was to investigate the potential associations between insulin resistance and serum concentrations of adiponectin and resistin, in young and older children from 4 countries: Japan, Thailand Italy, and the USA. Our data indicates that adiponectin and resistin vary by age, gender, and country of origin. We also found significant correlations between adiponectin levels and markers of insulin resistance. Although, we hypothesized that older US and Italian children would have higher insulin resistance, lower adiponectin, and higher resistin compared to Japanese and Thai children, our data did not confirm our initial hypothesis. Rather, our data indicates that Japanese and Thai children have lower adiponectin and higher resistin compared to Italian and American children. The lower levels of serum adiponectin concentrations that we found in Asian children could contribute to differences in expression of metabolic syndrome and its associated risk factors in Asian vs European and American children.

In this study, serum adiponectin concentrations in male and female children from all countries tended to be higher in younger compared to older children. Additionally, we found sex-based differences in adiponectin. Female children had higher adiponectin levels when compared to male children in both age groups. These tendencies remained seen after the adjustment for BMI or WC. Nishimura et al. [25] found, in a prospective study, that serum adiponectin levels were higher in 9 to 10-year-old boys and girls than in those aged 12–13 years old. Punthakee et al. [8] compared serum adiponectin levels of children aged 9, 13, and 16 years old, and found that adiponectin levels decrease as age increases, especially in boys. Nishizawa et al. [26] found that higher testosterone levels decrease plasma adiponectin levels and differences in the testosterone levels of boys and girls could be one reason for the difference in adiponectin levels between genders. They also showed that in mice [26], oophorectomy did not change adiponectin levels, but castration increased plasma adiponectin levels and that testosterone replacement subsequent to castration decreased plasma adiponectin levels [26]. The authors also noted that in their study, plasma adiponectin levels were significantly lower in men than in women [26]. As children progress through puberty, androgen levels rise, particularly in boys, and our data showing that adiponectin levels were lower in older Group 2 children than in younger Group 1 children as well as being lower in male children than in female children support these findings.

Given the higher prevalence of overweight and obesity among US and Italian children, we hypothesized that this group would have lower adiponectin compared with Japanese and Thailand children, however this was not supported by our findings. In our study, children from Japan and Thailand had lower serum adiponectin concentrations than those from Italy and the USA, suggesting that there are ethnic/racial differences in serum adiponectin between Asian and European/American children. These tendencies remained seen after adjustment for BMI or WC. Weyer et al. [24] reported that adiponectin in Pima Indians, a population at high risk of developing type 2 diabetes mellitus, was considerably lower than those in Caucasians. Similarly, Martin et al. [27] found that serum adiponectin was lower in South Asian than in Caucasian women, and the results of the Bogalusa Heart Study [28] showed that in both male and female children, serum adiponectin concentrations were lower in African-American compared to Caucasian children. Additionally, adiponectin was lower in male than in female Caucasian and African-American children [28]. In our study, we found differences in adiponectin among children from different countries which is consistent with findings in previous studies [27, 28].

In our study, serum resistin concentrations were higher in older Group 2 children. Like adiponectin, sex-based differences in resistin also existed. Female children had higher resistin compared with male children, especially in older Group 2 children aged 12–15 years. Additionally, serum resistin concentrations in male and female Japanese children were significantly higher than those from Italy and the USA, but lower than those in children from Thailand. Although these findings clearly differ from that of serum adiponectin, they still suggest that there are ethnic/racial differences with respect to the serum resistin in children from Asian countries compared with those in children from European/American countries. These trends were seen independently of adjustments for BMI or WC. Differences in resitin and adiponectin may be due to underlying variations in genetic markers. To our knowledge, this is the first report to show differences in serum resistin with respect to age, gender, and ethnicity within an international pediatric cohort.

We found significant correlations between serum adiponectin and fasting serum insulin levels and/or HOMA-IR as parameters associated with insulin resistance, especially in Group 1 female children and in most Group 2 male and female children. In multiple regression analyses serum adiponectin was associated with HOMA-IR in Group 2 male children from Italy and the USA and in Group 2 female children from all countries. Therefore, it is likely that adiponectin has a different role in the regulation of insulin resistance, depending on age, gender and ethnicity. The role of adiponectin in the development of insulin resistance might, thus, be more important in older than in younger children, in female than in male children, and in children from Europe and America than in children from Asian countries. Although serum adiponectin levels were higher in younger children than in older children, its role in insulin resistance may well become more important as children become older. When considering the age, gender, and ethnic/racial differences in serum adiponectin, as well as the fact that insulin resistance is predominantly found in European/American children in our study, these results may indicate that higher serum adiponectin concentrations might compensate for insulin resistance, particularly in older male children from Italy and the USA and in older female children from Italy.

Interestingly, across all groups, females also had higher insulin levels and HOMA-IR.

except in Thailand. As well, females had higher serum adiponectin concentrations than males in all age groups. Hoffman et al. [29] suggested increased insulin secretion in adolescent females increased perhaps due to lower insulin sensitivity in the periphery. As reported [26–28], differences in testosterone levels of boys and girls could be one reason for differences in adiponectin levels between genders.

Differences in adiponectin levels among children from different countries was consistent with findings in previous studies. Therefore insulin resistance with higher serum insulin levels and HOMA-IR may be modulated by higher serum adiponectin levels, especially in older females, as suggested by our multiple regression analyses. Nevertheless, other environmental factors should be taken into consideration, including food intake, physical activity [30, 31], and genetic characteristics, such as SNPs in the adiponectin gene [32]. These points need to be taken into consideration when addressing the higher HOMA-IR indices in US and Italian children despite their having, overall, higher serum adiponectin and lower resistin concentrations than the Asina cohorts. This divergence between HOMA-IR and adipocytokine concentrations, however, still needs further mechanistic study.

The relationship between resistin and insulin resistance has been controversial. In our study, multiple regression analyses showed that resistin was not associated with markers of insulin resistance in children from any of the countries. Osawa et al. [10] emphasized that SNPs of the resistin gene are strongly associated with insulin resistance in type 2 diabetes, whereas Li et al. [12] and de Luis et al. [13] detected no or only weak associations between resistin and insulin resistance in children and adults. Therefore insulin resistance markers such as HOMA-IR may be lower despite higher serum resistin levels in Japan and Thailand compared to Italy and USA especially in Group 2. It can be speculated that resistin might have another important role, for example, on the regulation of blood pressure, because univariate analysis showed that among children from all the countries, serum resistin level was partially correlated with various markers of metabolic syndrome, such as systolic and diastolic blood pressure. This premise is supported by the results of Takata et al. [33] who showed that resistin was positively correlated with blood pressure in type 2 diabetes patients and that it was independently associated with high blood pressure in adults with type 2 diabetes.

There were some limitations in our study. First, participants were divided into 2 age groups, but the data may be more easily interpreted if the participants were divided into subgroups according to the Tanner stage or serum levels of sex hormones, such as testosterone or estradiol. Because Tanner sexual maturity stage information was not collected, the age of our participants (Group 1: 8–11 years old, Group 2: 12–15 years old) may represent a mix of pre and early pubertal stages. Several studies have reported that the onset of pubertal testis and breast development varies among different racial/ethnic cohorts. For example, in Japan, Tanner stage 2 sexual maturity is between 9–11 years old among female and male Japanese children [34, 35]. Similarly, in Thailand, the US, and Italy, the age of pubertal onset can vary between 9.4-12.3 years of age [36–40]. Additionally, De Leonibus et al. recently reported that obese Italian females entered puberty at an early age (9.90 ± 0.78 vs. 10.32 ± 1.70, P = 0.016) when compared to non-obese female children [41]. Although our participants may have mixed pubertal stages, we did find consistent differences in insulin resistance, adiponectin, and resistin concentrations among groups and within each country suggesting that there are underlying age related differences which may be attributable to sexual maturation. Woo et al. [42] reported that serum adiponectin levels progressively decline through puberty, particularly in boys, and that the decrease of serum adiponectin is greater in non-lean boys. Bottner et al. [43] reported similar results in terms of the relationship between serum adiponectin levels and Tanner stage. Second, children from the USA were from mainly non African-American ethnic groups. Bush et al. [14] reported that adiponectin levels were lower in African-American children and adolescents than that in their Caucasian counterparts. Lastly, systematic biases may have been introduced because data was pooled from different cohorts which were initially a part of larger study designs. For example, in the USA, data was collected between 1994–1997 as part of the BioMarkers Study while in Japan, Italy, and Thailand, data was collected in 2002. Because of differences in study protocols, anthropometric markers such as blood pressure and waist circumference were measured differently among the four countries. Additionally certain parameters such as information on waist circumference was not available in Italy. Lack of funding prohibited laboratory analyses of high molecular weight adiponectin across all cohorts. However all blood samples were stored at −70 C and all laboratory analyses were centralized at a single core laboratory in Japan thereby minimizing biases with respect to measurement of serum concentrations of adiponectin and resistin, as well as other blood parameters.

## Conclusions

We found that serum adiponectin concentrations were or tended to be lower in children from Asian countries than in those from Europe and the US. Furthermore, adiponectin was higher in younger children and female children than in older children and in male children, respectively. We also found an association between adiponectin and insulin resistance among older female children for all countries and in older Italian and American male children. These findings suggest different roles for adiponectin in insulin resistance in different age, gender, and ethnic/racial groups. In contrast, serum resistin concentration seemed to be higher in children from Asian countries than in those from Europe and the USA but resistin had no or only weak associations with markers of insulin resistance. While our study is one of the few large international comparative studies demonstrating relationships between adipocytokines and factors associated with insulin resistance, further international comparative studies investigating genetic differences in adipocytokine levels and risk of insulin resistance are needed.

## Authors’ information

KT is an assistant professor and EI is a Professor of the Department of Pediatrics, Ehime University Graduate School of Medicine. All other co-authors are specialists within pediatrics and/or global health.

## Abbreviations

BMI: Body mass index
WC: Waist circumference
CVD: Cardiovascular diseases
HOMA-IR: Homeostasis model assessment of insulin resistance
SNPs: Single nucleotide polymorphisms
CHASE: Child Heart and Health Study
HDL-C: High-density lipoprotein-cholesterol
TG: Triglyceride
IDF: International Diabetes Federation
BP: Blood pressure
S.E.: Standard error
ANOVA: Analysis of variance
F.P.G.: Fasting plasma glucose.
